# Do People Use the Shortest Path? An Empirical Test of Wardrop’s First Principle

**DOI:** 10.1371/journal.pone.0134322

**Published:** 2015-08-12

**Authors:** Shanjiang Zhu, David Levinson

**Affiliations:** 1 Department of Civil, Environmental, and Infrastructure Engineering, George Mason University, 4400 University Drive, MS 6C1 Fairfax, Virginia, 22030, United States of America; 2 Department of Civil, Environmental, and Geo- Engineering, University of Minnesota, 500 Pillsbury Drive SE, Minneapolis, Minnesota, 55455, United States of America; Institut Pluridisciplinaire Hubert Curien, FRANCE

## Abstract

Most recent route choice models, following either the random utility maximization or rule-based paradigm, require explicit enumeration of feasible routes. The quality of model estimation and prediction is sensitive to the appropriateness of the consideration set. However, few empirical studies of revealed route characteristics have been reported in the literature. This study evaluates the widely applied shortest path assumption by evaluating routes followed by residents of the Minneapolis—St. Paul metropolitan area. Accurate Global Positioning System (GPS) and Geographic Information System (GIS) data were employed to reveal routes people used over an eight to thirteen week period. Most people did not choose the shortest path. Using three weeks of that data, we find that current route choice set generation algorithms do not reveal the majority of paths that individuals took. Findings from this study may guide future efforts in building better route choice models.

## Introduction

Route choice analysis investigates the path travelers follow to implement their travel plans. It is the most frequent, and thus arguably the most important decision travelers make on a daily basis. Empirical studies show that route and schedule changes are the most dominant reactions to network changes [1, 2]. Any sound arguments for infrastructure initiatives or policy changes must be built on precise and reliable prediction of link flow and travel time, and thus on underlying route choice decisions. Travelers differ in attributes (value of time (VOT), willingness to pay, time budgets, etc.), behavioral preferences (e.g. willingness to take risk, willingness to switch routes with potential savings) experience, and knowledge about travel, all of which could lead to significant heterogeneity in route choice behavior. Mainstream research and practice, however, has treated trips as the unit of analysis since the 1950s. This trip-based modeling paradigm assumes homogeneous travelers and tends to focus on some form of User Equilibrium (UE) state, in which “the journey times in all routes actually used are equal and less than those which would be experienced by a single vehicle on any unused route” (known as Wardrop’s first principle [3]).

Although this shortest-path (usually measured as shortest travel time path) assumption and the resulting aggregate UE approach are simple, intuitive, and easy to implement (efficient solutions are widely available), it has been criticized for ignoring the heterogeneity among travelers and limitations in their spatial knowledge and reasoning ability. Empirical studies (e.g. [4–6] among others) on route choice criteria based on stated preferences indicate that many other factors such as distance and reliability also affect route decisions. These factors, together with the imperfections in travelers’ perception and reasoning capacity, can push travelers away from the shortest time route. However, Wardrop’s first principle has rarely been directly tested in the field. A pilot study by Jan et al.[7] concluded that travelers often chose paths that differed significantly from the shortest time path. Limited by the number of samples (83), they did not answer a natural follow-up question: to what extent the actual routes deviate from the shortest time path, which has significant implications for research aiming to relax these behavioral restrictions on UE models.

Some researchers argue that travelers are still rational in minimizing their perceived travel time while limited in their capacity to get perfect information. Daganzo and Sheffi [8] introduced the Stochastic User Equilibrium (SUE) model and used a random component to represent the perception error and other randomness in the system. Other researchers further pointed out that many irrational components could be perfectly rational if modelers could capture travelers’ preferences more accurately. Random Utility Maximization (RUM) models have been applied to investigate a wider spectrum of route choice preferences (e.g. shorter distance, less toll, or more freeway use). Bekhor et al. [9] investigated 16 possible combinations of plausible preferences. Along this research branch, significant efforts have been dedicated to overcome the so-called Independence of Irrelevant Alternatives (IIA) problem. Many variations of RUM models have been introduced (e.g. C-logit model [10], Path-Size logit [11], among others). With few exceptions (e.g. [12]), most of these models require explicit enumeration of all feasible routes (the consideration set). A consideration set either too large or too small could significantly compromise flow prediction [13]. Because route enumeration is also time consuming, it is crucial to establish the scope of routes that are likely to be used by travelers.

In parallel with the research efforts on RUM models, other researchers treat travelers as “satisficers” who will switch to a more attractive route when the benefits such as time savings are large enough. There has been abundant literature on boundedly rational users in fields such as psychology and economics. Knight [14] pointed out that “the rational thing to do is to be irrational” when considering the “deliberation and estimation cost”. Conlisk [15] provided a comprehensive review on this topic and concluded that the importance of bounded rationality is supported by both wide-ranging evidence and its excellent success in describing behavior. In the context of travel demand modeling, Mahmassani and Chang [16] introduced the Boundedly Rational User Equilibrium (BRUE) when investigating the departure time choice on an idealized network with a single Origin-Destination (OD) pair and a unique route. Nakayama et al. [17] employed a micro-simulation model to investigate BRUE and pointed out that BRUE does not necessarily converge to UE. Lou et al. [18] further evaluated the mathematical properties of BRUE and revealed that BRUE assignments are not unique and when the threshold for route switching is large enough, congestion pricing schemes may make the system worse off. Their results showed that the extent to which people’s choices deviate from the shortest path is also crucial in determining the domain of possible BRUE results, and thus performance of policies under evaluation.

In contrast with the aggregate modeling paradigm, many researchers argue that the route choice problem should be investigated from an individual perspective. For example, Tawfik and Rakha [19] argued that a paradigm shift from network (such as UE and SUE) to driver oriented modeling is necessary for the route-choice problem and revealed the route choice patterns estimated by the two modeling paradigms are very different based on data collected in a real-world route choice experiment. Examples of the latter category includes cognitive-psychology models [20], fuzzy models [21], and models based on data mining [22], among others. Zhang developed a behavioral user equilibrium model [23].

However, many advanced models in both categories still heavily rely on the shortest path assumption. For example, Jha et al. [24] assume drivers are provided with travel time information on five shortest paths when investigating day-to-day dynamics using DYNASMART. Some models such as MATSIM [25] applied RUM models on a pre-determined choice set generated by the K-shortest path algorithms [26]. The number of alternatives and their generation process is arbitrary and depends on computing convenience. Similarly, route choice models which adopt a rule-based paradigm (e.g.ALBATROSS by Arentze and Timmermans [27] and Agent-based Route Choice model by Zhang et al. [28]) also require a well-defined consideration set based on which route choice rules could be derived and applied for flow prediction. A choice set which is not correctly specified can distort both the calibration and the prediction process [13]. Therefore, the research question, “How Far is Traffic from User Equilibrium?”, has significant implications for both macroscopic and individual travel demand models.

However, empirical studies about formation of the consideration route set are few (Bovy [13] reviewed this topic). The difficulties are three-fold: first, unlike other dimensions such as mode and destination choice, route choice is very difficult to describe. Bekhor et al. [9] reported gaps and ambiguity in the written descriptions of travelers’ habitual route in their analysis and shortest paths were applied to fix them. Second, most data only contain habitual routes, while a large portion of candidate routes that have been considered but not frequently chosen have not been reported during the survey process. Third, the size and complexity of the regional traffic network makes it difficult to define and distinguish highly overlapping candidates. Prato [29] later provided a more comprehensive review (in both choice set generation and discrete choice model estimation) and arrived at similar conclusions.

Bekhor et al. [9] evaluated the habitual route reported by faculty and staff from the Massachusetts Institute of Technology in Cambridge, Massachusetts. The data were collected in written description and shortest paths were utilized where ambiguity or holes were found. Among the 188 routes between 91 OD-pairs, 37% of respondents followed the shortest time path (90% overlapping is required for coverage) and 22% followed the shortest distance path. Similarly, Prato and Bekhor [30] evaluated 236 routes between 182 OD pairs in Turin, Italy and found 53.5% of respondents chose the shortest distance path while 43.3% chose the shortest time path. However, neither study reported how much longer those non-shortest paths were compared to the shortest ones. Moreover, both studies used planning network and assignment results to derive path travel time, which may not be sufficiently accurate.

Spissu et al. [31] show significant amounts of intra-individual variability from a sample of 12 university students. Related research explained route switching behavior as a function of factors beyond travel time [32].

Several studies have examined taxis. Morikawa et al. [33] measured the travel time on 6 alternative routes (one of them a toll road) based on Global Positioning System (GPS) data from 1500 taxis in Nagoya, Japan during two months and evaluated the route choice decisions of the same set of vehicles. They found a high percentage of trips employed non-shortest path (e.g. using the toll road while travel time on it is even longer than toll-free alternatives) and they concluded that this phenomenon is due to the lack of knowledge of network connectivity or travel time reliability. However, their samples are biased in both the subjects identity (taxi only) and the trip destination (the airport). Ding et al. [34] investigated routing policy choice set generation based on individual-level route choice data from GPS observations in Stockholm, Sweden and Singapore. They found that with a threshold of 80% to define the same route, a combination of link elimination and simulation method can identify 92% of the route people actually use. Ma and Fukuda [35] compare shortest path routes of taxis with hyperpaths and find that hyperpaths have more explanatory power. GPS data from Taxis may not be consistent with general traffic.

A branch of the literature has explored the route choice decisions of users of non-auto modes. Bicyclists are known not to follow the shortest path because of obvious qualitative differences in routes. This has included stated preference work [36] as well as more recent GPS based revealed preference analysis [37–40]. Motorcyclists also have different preferences [41].

The onset of recent smart phone based GPS (as well as earlier research based on triangulation) has opened new data avenues that promise to make this type of analysis more widespread [42]. Similarly, new tools for both Geographic Information System (GIS) and route choice modeling are also making this research more feasible [29, 43, 44].

Empirical research demonstrates that automobile travelers care about tolls [45], stops [46], reliability [47], traffic lights [48], and aesthetics [49]. Further, travel time is systematically misperceived [50].

This study investigates the characteristics of routes followed by randomly recruited subjects during a study period of up to 13 weeks and compares them with the shortest time or distance path. GPS devices have been installed in subjects’ vehicles and their trajectories during the entire study period documented. The travel trajectories of instrumented vehicles are projected onto a GIS map of high-resolution and their characteristics analyzed. In contrast with previous studies, this study uses observed travel time derived from the travel time of the same set of instrumented vehicles (serving as probe vehicles). The shortest time or distance paths are developed based on the same GIS map, ensuring the consistency across different routes. Findings from this study could help evaluate current route choice models and provide guidance for constructing choice sets. Moreover, the shortest path assumption is still commonly employed in practice, from aggregate planning models to emerging agent-based models (e.g. TRANSIMS [51], MATSIM [25]). Therefore, an empirical evaluation of how far this assumption deviates from observation in the field would help researchers and practitioners better interpret results and improve future models.

The next section summarizes major findings in this study. Details of modeling approach and data used in this study will then be presented.

## Results

This study captures the actual routes travelers follow during an eight-week study period using GPS data detailed GIS road map. The actual routes are then compared with the shortest time path predicted by the model, showing the gap between the ideal assumption of human route choice behavior and the actual behavior. Four popular choice set generation algorithms, all of which are widely used in modeling human route choices, are then evaluated using the identified routes. Details about the data and the way routes are identified will be presented in the Materials and Methods section.

### Do people use the shortest path?

**Fig 1:**
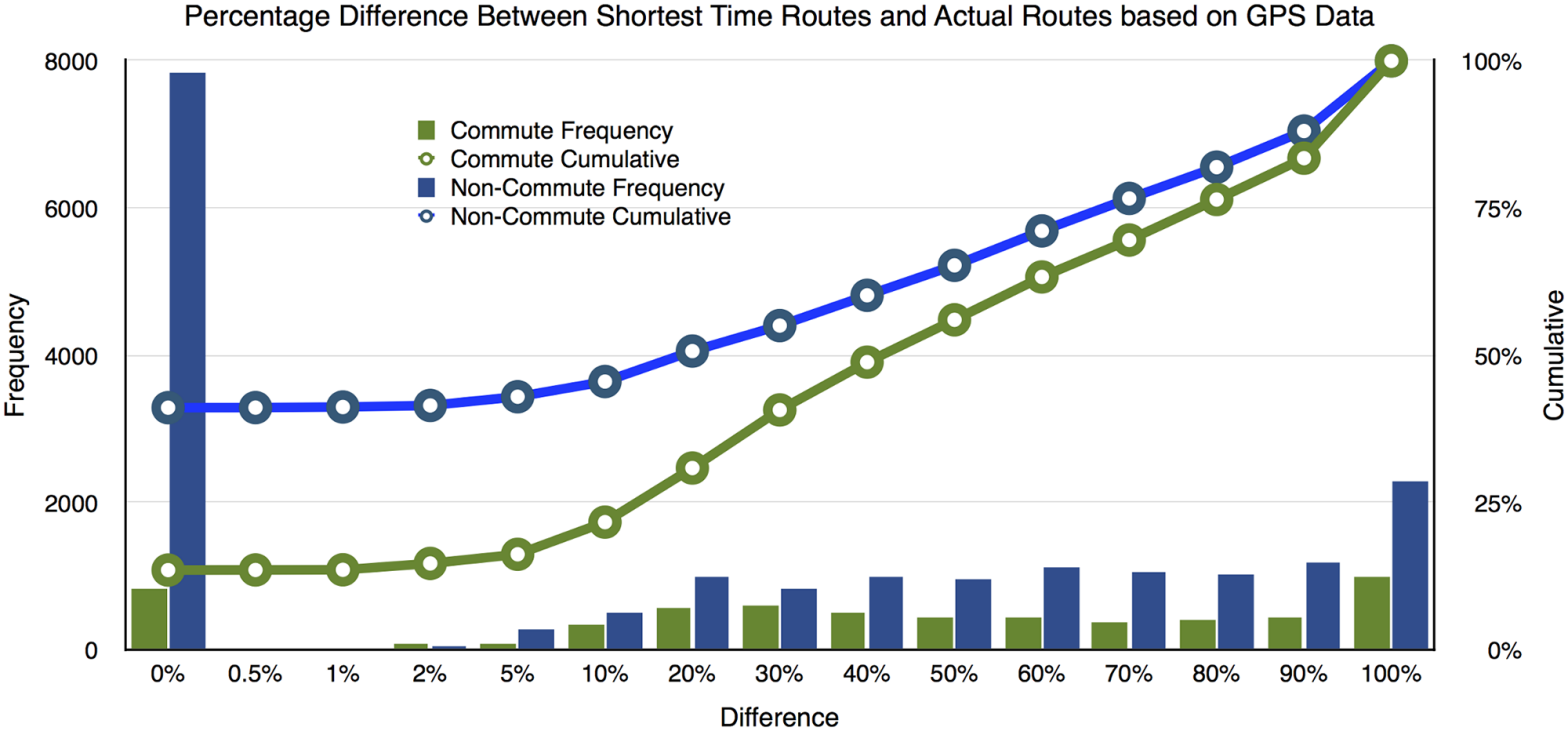
Difference between GPS-revealed route and the shortest time route between the same OD.

The identified routes and the shortest time routes are then compared segment-by-segment using GIS and the results are summarized in Fig 1 for commute and non-commute trips. If two routes overlap, the difference should be 0. In contrast, if two routes do not overlap at all, they are 100% different in the graph. By the most strict standard (0% different), about 34% of all trips (commute plus non-commute) followed the shortest time path. If the standard is relaxed to 10% as most previous studies suggest, then about 40% of all trips follow the shortest time path.

This result may seem high because a large number of short trips do not actually have many feasible alternatives and thus are more likely to follow the shortest time path. If we only evaluate commute trips which are usually longer, then only 13.5% of trips completely coincide with the shortest time path. By relaxing the standard to 10% overlapping, 21.7% of trips followed the shortest time path. Non-commute trips are similar to the overall set of trips as most trips are for non-work purposes.

**Fig 2:**
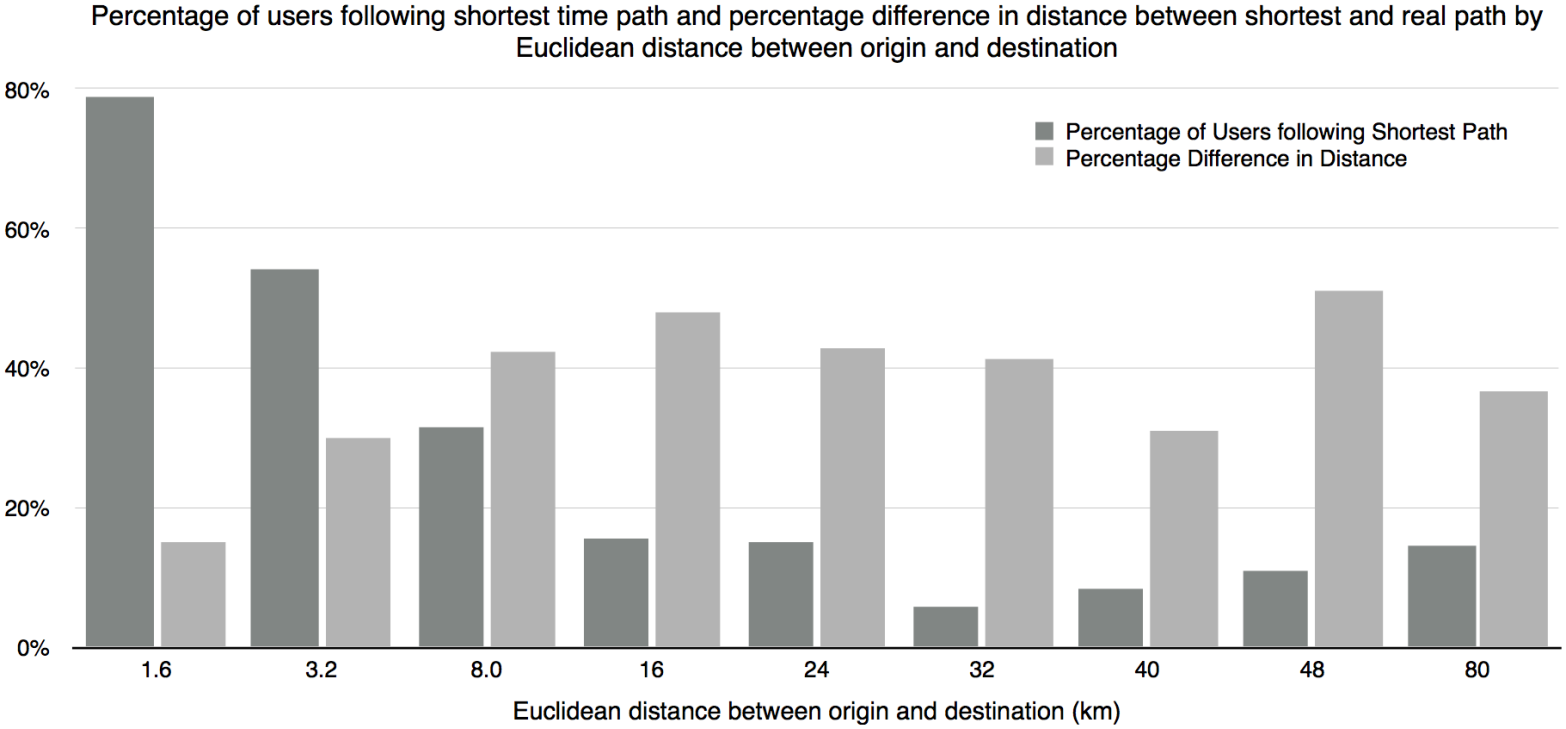
Percentage of trips in which travelers follow the shortest time path and percentage difference in length between the actual route and the shortest path by Euclidean distance between the origin and destination.

Route choice behavior could be constrained by availability of meaningful alternatives. For example, when the trip is very short, the entire trip could be served by only one straight road and any alternative route would incur a significant detour. At the other extreme, when the travel distance is very long, the optimal choice could be to access the freeway at the entrance closest to the origin and get off at the exit closest to the destination. Therefore, people are more likely to follow the shortest path at both extremes. Fig 2 shows the percentage of travelers following the shortest time path by different Euclidean distance between the origin and the destination for that specific trip. As we will discuss later, there is no universally accepted threshold to define two similar routes as different. Fig 1 showed that 2% could be a natural cut. Therefore, we classify people who deviate from the shortest time path by less than 2% in length as those following the shortest time path, although other thresholds could also be used. The results in Fig 2 are consistent with our previous analysis. Travelers are more likely to follow the shortest path when the trip is short. The percentage goes down quickly as the trip becomes longer. But it picks up slightly again when the trip becomes very long. In our case, the percentage reaches the lowest (around 5%) when the air distance between the origin and the destination is around 20 miles.

Fig 2 further summarizes the length of the portion where actual routes deviate from the shortest routes normalized by the length of the actual routes. Each bar shows the average percentage number for all the routes with the Euclidean distance between trip origin and destination falling within the corresponding bin. The overall trend is consistent with our hypothesis except for the extreme long trips (≥ 40 *km*). Due to the low density of the freeway network, the route choice for long trips could significant deviate from each other if the traveler chooses different initial movement (e.g. first go East vs. go South). It is also complicated by the existence of the Mississippi River and the limited number of river crossing points (people may prefer different river crossing points, especially after the I-35W collapse). Empirical study on a different network could help to test these hypotheses.

**Fig 3:**
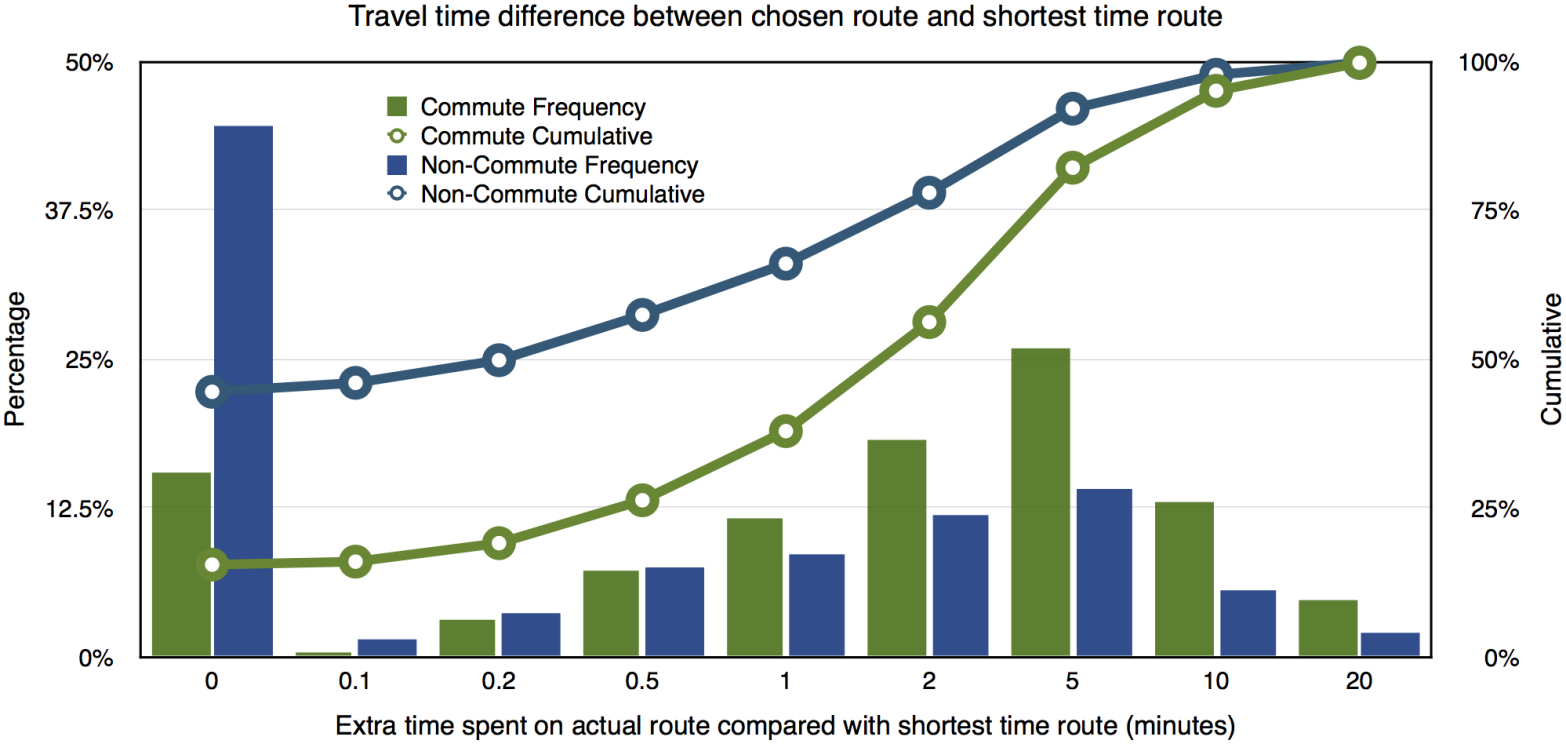
Comparison in travel time between actual commute/non-commute trip route and corresponding shortest time route.

The theory of Boundedly Rational User Equilibrium argues that people can choose any of the alternative routes whose travel time does not exceed the shortest time route by an empirically defined threshold. Therefore, it is interesting to compare the travel time on route people actually choose and the shortest time route. Fig 3 summarizes the results. For about 50% of the trips, the actual chosen routes that are less than 30 seconds longer than the shortest time routes. In almost 90% of cases people choose routes that are less than 5 minutes longer than the shortest time routes. Commute routes deviate from shortest time routes slightly more in percentage compared with non-commute routes. The difference between actual routes and shortest travel time routes for most trips is small, but non-trivial, since 5 minutes represents almost one-fifth of the average commute time (24 minutes) in the Twin Cities area.

**Fig 4:**
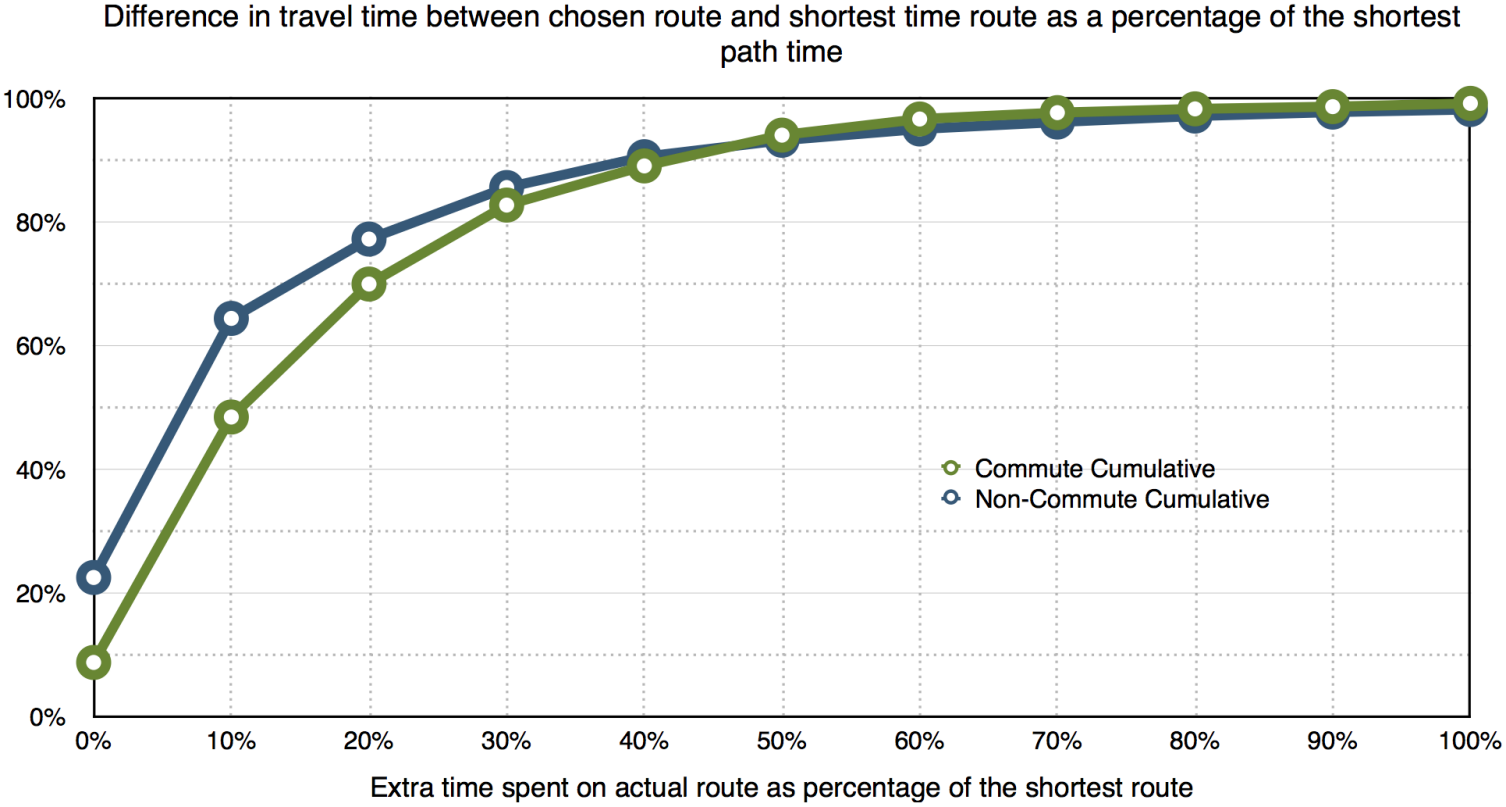
Difference in travel time between chosen route and shortest time route as a percentage of the shortest path time.

The relevant scale in difference may also provide important empirical evidence. Fig 4 shows the difference in travel time between the chosen and shortest time route as a percentage of the shortest path time. About 55% of non-commute and 30% of commute trips follow a route that is almost as good as the shortest time path (less than 5% longer in time). Although about 80% of non-commute trips and 70% of commute trips follow a route that has a travel time less than 20% longer than the shortest time path, the number of trips that follow a much longer route is still significant. People may have different motivations in choosing a route other than the shortest. For example, they may drop off children or spouse (in ways undetected by our definition of trips as engine off or being stopped for a sufficiently long time), or stop briefly for a coffee, or simply because they prefer to use a route that possesses other desirable features. The empirical evidence presented in this paper pointed out such non-optimal choices from traffic assignment perspective is not trivial either in frequency or in significance. More empirical studies are warranted to better understand such behavior.

### Route choice set generation

Choice set generation plays an important role in route choice since many existing models require explicit enumeration of the routes to be considered. Extending previous research (such as [9, 30]), this study evaluates 4 widely-used route choice set generation (K-shortest path) algorithms based on the same GPS data as described in previous sections. By using the GPS data instead of surveys, this study avoids gaps and ambiguity when identifying routes people actually use. Moreover, many people use more than one route between the same origin and destination within a time period, which are usually under-reported in a one-day, or even multi-day, survey. In contrast, this diversity in route choices can be easily captured by GPS data and applied for testing choice set generation algorithms. Therefore, an evaluation of choice set generation algorithms based on GPS data should inform travel demand modeling.

To simplify the problem, the current study only looks at home-to-work trips before the opening of I-35W replacement bridge, when traffic conditions are stable. The same analysis could also be applied for trips of other purposes and for other time periods. In total, 657 home-to-work trips made by 95 subjects have been identified during these three weeks. Any home-to-work trips that included identifiable side stops are excluded from this study. Four algorithms are evaluated here:

1. Link labeling: The labeling approach, originally proposed by Ben-Akiva et al. [52], calculates paths that maximize different definitions, including shortest travel time, shortest free-flow travel time, shortest distance, least congestion, etc.
2. Link elimination: The link elimination algorithm [53] generates *K* ^*th*^ shortest path by finding the shortest path after removing all links of the first *K* − 1 shortest paths from the network. It stops when no new path can be found because of missing network connectivity. This study adopts a variant of this algorithm by only eliminating one-third of links (those located in the middle one-third of the path) during each iteration to avoid premature stopping due to failures of network connectivity when major junctions are removed.
3. Link penalty: The link penalty approach [54] generates new shortest paths after multiplying the travel time of each link on the current shortest path by 1.05.
4. Simulation: The simulation approach computes the shortest path for each draw of link impedances. Many impedance distributions have been tested and three sets of them are presented:
  (a) normal distributions with mean travel time and travel time variance derived from GPS observations,
  (b) normal distribution with mean travel time derived from GPS observation and a variance equal to 15% of the mean (the optimal parameter with an overlap threshold of 80%), and
  (c) normal distribution with mean travel time derived from GPS observation and a variance equal to 20% of the mean (which has been used in [30]).
  A left truncation that is equivalent to a minimal speed of 8 mph (12.9 *kmh* ^− 1^) is adopted. Sensitivity tests to the number of draws are also applied. Table 1 summarizes the percentage of routes that are covered by each algorithm again all routes observed through GPS data. The random draw of the travel time on each link is independent, though there is evidence that strong correlation exists between the travel times on links [55].

**Table 1:** Coverage: Percentage of generated routes which are observed using alternative route choice set generation algorithms based on GPS data and Twin Cities regional planning network.

	Overlap threshold (%)		
Algorithm description and parameters	100	90	80
Labeling approach			
Least time	2	9	16
Least free-flow time	6	16	23
Least distance	2	5	9
Maximize freeways path	4	12	19
Minimize(*Freeway* + 2*Expressway* + 4*Arterial* + 4*Local*) Time			
Minimize freeways path	1	2	3
Minimize(4*Freeway* + 2*Expressway* + *Arterial* + *Local*) Time			
All labels combined	9	25	37
Link elimination for Least Time path	3	11	25
(eliminate 33% of middle links)			
Link penalty 15 unique routes	6	24	44
Link penalty 40 unique routes	7	28	50
Link penalty 80 unique routes	8	28	50
Minimize simulated time, observed *σ* ^2^, 12 draws	4	13	25
Minimize simulated time, observed *σ* ^2^, 24 draws	6	15	28
Minimize simulated time, observed *σ* ^2^, 48 draws	7	18	33
Minimize simulated time, *σ* ^2^ = 15% of mean, 12 draws	2	13	31
Minimize simulated time, *σ* ^2^ = 15% of mean, 24 draws	4	20	39
Minimize simulated time, *σ* ^2^ = 15% of mean, 48 draws	5	23	44
Minimize simulated time, *σ* ^2^ = 15% of mean, 96 draws	10	30	59
Minimize simulated time, *σ* ^2^ = 15% of mean, 192 draws	12	39	63
Minimize simulated time, *σ* ^2^ = 20% of mean, 12 draws	2	10	28
Minimize simulated time, *σ* ^2^ = 20% of mean, 24 draws	4	16	39
Minimize simulated time, *σ* ^2^ = 20% of mean, 48 draws	5	23	46
Minimize simulated time, *σ* ^2^ = 20% of mean, 96 draws	10	33	57
Minimize simulated time, *σ* ^2^ = 20% of mean, 192 draws	15	44	60
Total number of observed routes (counts)	249	189	163

To avoid trivial alternatives, overlap thresholds must be defined when comparing different routes. A wide range of values have been used in literature and no consensus about appropriate thresholds has emerged. This study tests three different thresholds of the percentage of links in a path that are identical between the observed and generated route, 100%, 90%, and 80%. The total number of observed routes increases as we adopt a higher threshold to distinguish different routes. Overall, 249 routes have been observed if two routes are only the same when 100% of their link segments overlap.

As Table 1 shows, no single label performs well in predicting routes people actually use. The coverage provided by the least time approach (9% with an overlap threshold of 90%) is consistent with previous analysis on the percentage of commuters who follow the shortest path. The coverage is lower than that found by Prato and Bekhor [30] based on data collected in Turin, Italy (about 27%). However, the Turin network only contains 1,427 links, while the Twin Cities network contains 22,477 links. The extra complexity introduced by network size makes it harder to generate routes people actually use on the Twin Cities network. Most travelers prefer using freeways to local streets. And travel time is in general a better impedance measure than distance. When combining all five labels together, the labeling approach can only generate 37% of all observed routes with an overlap threshold of 80%.

The link elimination algorithm generates between 29 and 58 unique routes (with an overlap threshold of 90%) for subjects. However, it could eliminate crucial links of observed routes in the first few iterations, preventing us from replicating those routes through an iterative path searching process. Therefore, it is not surprising to find a very low coverage rate (11%) for the link elimination algorithm.

The link penalty algorithm performs better in general. However, increasing the required number of unique routes before the algorithm stops does not significantly improve the coverage rate (from 24% to 28% when required unique routes increase from 15 to 40 (the number of routes is consistent with that used in Bekhor et al. [9])). The algorithm could have continued to identify similar routes with relatively small differences. Choosing an appropriate penalty factor could be crucial for improving efficiency of the algorithm, which presents an interesting topic for future research.

The simulation approach outperforms its deterministic counterparts. The coverage rate is only about one-third if we generate travel time from observed travel time distributions. By choosing a travel time distribution with relatively large variance (observed *σ* ^2^ is on average 3% of the mean on freeway links and 14% of the mean on arterial links), we can generate as many as 63% of routes observed through the GPS study in 192 iterations. A variance which is too large will also result in a lower coverage or hit ratio. A variance equal to 15% of the mean provides the highest coverage or hit ratio at a 80% overlap threshold, or a small number of draws at a 80% overlap threshold. However with more draws and at the 90% overlap threshold, a different variance may have a higher hit ratio. We can expect higher coverage through more iterations. However, the marginal benefit of doing so diminishes as the number of iterations increases. In addition to its advantage in explicitly generating choice set, the simulation approach can also be applied on network loading, which defined the feasible choice set implicitly. For example, Mirchandani and Soroush [56] applied the simulation approach to solve the generalized traffic equilibrium with profitabilities link travel time and heterogenous perceptions.

Running time for the link elimination, link penalty, and simulation approaches are similar, while minimizing one label for the labeling approach takes much less time (a few seconds). The link elimination approach stops in 1636 seconds. The link penalty approach with a stop criterion of 80 unique routes for each OD takes 2248 seconds. The simulation approach finishes 192 iterations in 1291 seconds (all tests are conducted on a Power Mac G5 3.00GHz). Considering the number of observed routes each approach can generate, the simulation approach exhibits significant advantage.

### Conclusions

This study empirically tests, and rejects, the shortest-path assumption which has been widely applied in both research and practice. The results show that about two-thirds of the subjects do not use the shortest travel time path during a three week study time period. No subjects followed the shortest distance path unless it also coincided with the shortest travel time path.

None of the existing route choice set generation algorithms provides satisfactory results in generating a choice set. Most of these algorithms rely on evaluation of shortest time paths. However, travelers clearly have other preferences when making their route choices. Therefore, a better understanding of people’s route preferences could also inform the development of choice set generation algorithms. Clearly, a choice set that includes all alternative routes would in turn contribute to improvement in the accuracy of individual route choice modeling. The GPS-based approach developed in this study reveals people’s day-to-day route patterns with an accuracy that cannot be achieved through conventional surveys. The multiplicity of routes between the same origin and destination becomes obvious when using GPS data over a long period of time, which poses new challenges for choice set generation algorithms. The coverage rates provided by all algorithms evaluated in this study are consistently low. To cover most routes that people may choose, a wide spectrum of preference labels should be considered. Although simulation approaches do not directly address diversity in route preferences, they may actually reflect imperfections of network knowledge and randomness in behavior. Consistent with previous studies, this study finds that simulation approaches provide an efficient way to generate alternative routes and outperforms many deterministic route generation algorithms.

In most circumstances, people choose routes that are less than 5 minutes longer than the shortest time routes. However, we have observed some trips that represent a significant detour from the shortest time path. Since trips in this study are defined based on the engine-on and engine-off activities or dwell time, we cannot exclude the situations when people detour for purposes such as dropping off passengers. These side trips could help to explain the occasional unusually long detours observed in our data, especially for commute trips. GPS data alone cannot provide information about trip purpose. More advanced analyses that combine GPS data with land use data to identify trip purposes can be used [57].

## Materials and Methods

This study has been reviewed and approved by the Institutional Review Board (IRB Code Number 0806S34983) of the University of Minnesota. All participants have been informed about the study, and been asked to read and sign the consent form before their participation. This material is based in part upon work supported by the National Science Foundation under Grant No. 0825768, BRIDGE: Behavioral Response to the I-35W Disruption: Gauging Equilibration; Minnesota Department of Transportation project Traffic Flow and Road User Impacts of the Collapse of the I-35W Bridge over the Mississippi River; Oregon Transportation Research and Education Consortium for the project Value of Reliability; and the University of Minnesota Metropolitan Consortium. No additional external funding received for this study.

### GPS Data

GPS data utilized in this study was collected during a 13-week long study targeting behavioral reactions to the I-35W Bridge reopening on September 18th, 2008. Recruiting occurred via announcements on craigslist, City Pages online and newspaper (a local free weekly), flyers at grocery stores and local libraries, postcards handed out in downtown parking ramps, and email to more than 7000 University of Minnesota staff (excluding students and faculty). People interested in participating in the study completed an on-line survey, providing background information about demographics, driving habits, job and residential locations, and commute routes before and after the I-35W bridge collapse. Participants were randomly selected among those who 1) were between 21 and 65 years old, 2) commute alone, 3) have a valid drivers license, 4) are likely to be affected directly or indirectly by the opening of the new I-35W Bridge according to their usual commute routes. Either a logging Global Positioning System (GPS) devices (QSTARZ BT-Q1000p GPS Travel Recorder powered by DC output from in-vehicle cigarette lighter) or a real-time communicating GPS device (adapted from the system deployed in the Commute Atlanta study ([58]) was installed in the vehicle of study participants. The GPS device is non-intrusive and unlikely to affect the behavior of participants. No instructions were given and participants were free to make travel choices. In total, 190 subjects participated in this study. However, only 143 GPS records were recovered due to the failure of devices (the data from GPS loggers could only be checked at the end of the study. Some of them failed because of power supply problems, such as being disconnected by subjects).

The logging GPS devices accurately monitored the travel trajectories of each probe vehicle at a frequency of one point per 25 meters up to 13 weeks, about 3 weeks before the reopening of the bridge and between 8 and 10 weeks after it. The real-time communicating GPS device recorded the position of instrumented vehicles for every second. The geographic location and time stamps of each point were documented and projected onto a GIS map for post-processing. The GPS data were then matched to The Lawrence Group (TLG) Twin Cities network, a detailed network conflated to the real road geometry, using ArcGIS [59].

An algorithm was developed and applied to ensure all points have been snapped to the nearest link which

- is directly connected to the upstream link previously identified,
- is consistent with the travel direction of nearby GPS points, and
- is connected to the downstream link which is also consistent with travel direction of downstream GPS points.

This algorithm rules out the possibility of incorrectly snapping the GPS point to the link on the opposite direction and on crossing directions. The high resolution of one point every 25 meters (the real-time communicating GPS provided an even higher resolution) reduces the possible of holes and discontinuity in identified routes to a minimum. In rare cases of data losses due to the communication difficulties with satellites, the shortest time path was used to connect the different segments of the same trip. This algorithm, combined with accurate GIS files, ensures that the right links will be identified for each trip. It also helps to ensure that the speed estimated from vehicle trajectories will later be assigned to the link through which travelers passed. A visual check was conducted for all trips of two random subjects during the entire study period, and confirmed the accuracy of the algorithm.

### Link Speed Estimation

The speed with which the probe vehicle traversed a link along its trajectory could be estimated by comparing the spatial and temporal distances between points at each end of the link. The average link speed could be estimated from all probe vehicles passing this link during a defined time period. There has been a large body of literature discussing the minimal number of observations required to ensure reliable speed estimates. For example, Cheu et al. [60] concluded that ten probe vehicles must pass though a link within the sampling period to achieve an accuracy within a 95% confidence interval. The number of observations required for a reliable travel time estimate depends on speed variance on each link and the desired confidence level. For this study, a link speed estimate was regarded as valid only if more than 10 samples were available during that time period.

**Fig 5:**
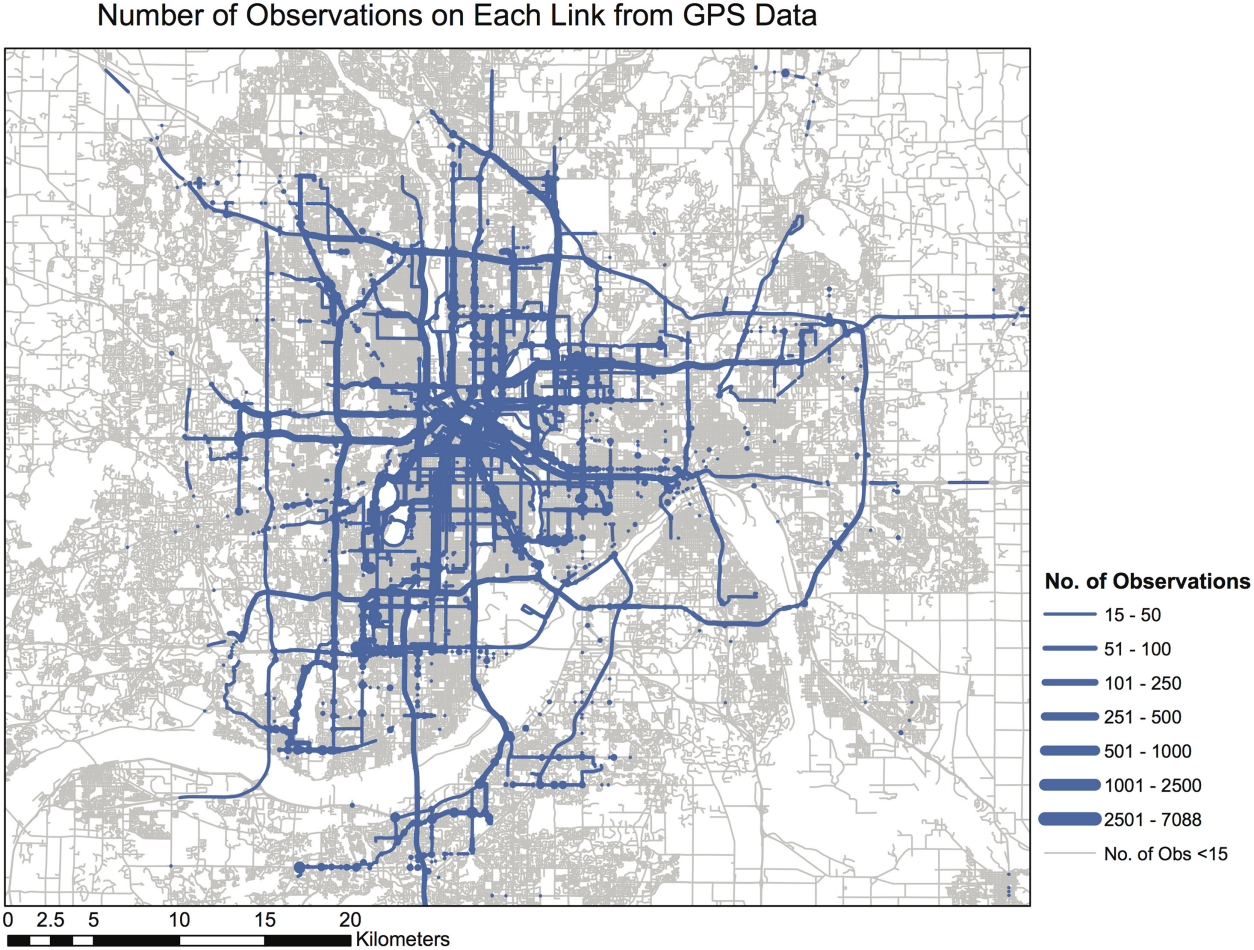
The number of speed observations on each link during the entire study period.

The large number of GIS equipped vehicles act as probe vehicles for the purpose of measuring travel speed on the network. The long study period allows us a large number of observations not only on freeway links, but also on major arterial links and local streets in downtown (see Fig 5). The latter is very important since it represents a significant chunk of total traffic and is unavailable in previous studies relying upon freeway loop detectors. Speed samples on arterial roads in the outer suburbs are generally low. However, road density in those areas were low and the traffic was unlikely to vary much due to scattered demand. Therefore, speed on roads with insufficient samples were assumed constant through the study and equal to the average speed on all the links of the same functional class defined by the US Census Bureau in their TIGER files [61]. The data can be downloaded from http://www.datafinder.org

Two major network changes occurred during this study time period: the replacement I-35W bridge was opened on September 18, 2008 and a section of the fourth lane westbound on the I-94 Bridge between the interchange with I-35W and Highway 280, a major mitigation measure implemented after the bridge collapse in 2007, was closed on October 12, 2008 and returned to operation as a bus-only shoulder lane.

Parthasarathi and Levinson [62] investigated the speed pattern for the Twin Cities based on both travel survey and loop detector data, and concluded that the morning and the afternoon peak periods (when congestion is sufficient to affect speed) are 6:00 am to 9:00 am and 2:00 pm to 7:00 pm, respectively. Combining the three time-of-day periods, Morning Peak, Middle of the Day, and Afternoon Peak, with the three phases, August 26th—September 18th, September 18th—October 12th, and October 12th—November 30th, 9 study periods were defined. All speed observations during non-holiday weekdays were pooled for each time period accordingly and average speed for each link with more than 10 samples was estimated. Because of their minor role in traffic analysis and the small number of observations available, two other time periods, Before Morning Peak and After Afternoon Peak, were ignored in this study.

The TLG network contains 290,231 links and 113,864 nodes for the Twin Cities Metropolitan area. Although it provides great accuracy, it dramatically slows down the path search algorithm. Moreover, the amount of observed data on suburban streets are low and trip variation in these regions is also low due to low road density. Therefore, the metropolitan planning network, which contains 22477 links and 8618 nodes and has been widely used in regional traffic analysis, has been used in the analysis. Speed estimation derived on the TLG network was transferred to the overlapping link from the planning network. Both networks have been conflated to the same geometry and overlapped accurately. Bovy [13] and Schuessler et al. [63] discuss network simplification.

### Commute and non-commute trips based on GPS Data

By following the steps described in the second section, links are identified along the vehicle trajectories, which are then divided into trips. A trip is defined between one engine-on and engine-off event, or when the vehicle failed to move more than 25 meters within 5 minutes (dwell time), or when the vehicle deviates from street center lines for more than 20 meters during 5 minutes, whichever comes first. The length of dwell time used to define trip ends in previous studies varies from 45 seconds to 300 seconds (e.g. 45s [64], 120s [65, 66], 300s [67] or multiple values [68]). The upper bound has been chosen in this study because 1) the ramp metering system in the Twin Cities area generates queues with a maximum delay of 4 minutes at freeway on-ramps; 2) a typical cycle length at signal-control intersections is 180s and it is possible to wait more than 1 cycle during peak periods; 3) the accuracy of the GIS map has helped to identify short off-street stops. Other filtering criteria such as circuity [69] and headway change. Du and Aultman-Hall [68] have also been suggested. However, the effectiveness of these criteria depend on the characteristics of the GPS data and need to be validated against detailed travel diary data. Therefore, this study did not adopt these more context-specific rules.

All participants in this study were frequent commuters. A large number of trips could be observed. This is important because it increases the chance to identify the commute routes that are seldom used but do exist in the consideration set, which cannot be revealed by reported habitual routes used in previous studies. We separate commute routes and non-work routes because they are likely to differ in time pattern; further commute trips usually have a targeted arrival time while non-work trips tend to be more flexible.

Home-to-work trips are defined as any trips starting within a 600 m radius from home and ending in a 600 m radius from the work location during a work day, without any stop longer than 5 minutes. The work-to-home trips are defined similarly. The threshold of 600 meters represents approximately 4 city blocks, which is chosen by observing parking and work places for a subset of subjects.

**Fig 6:**
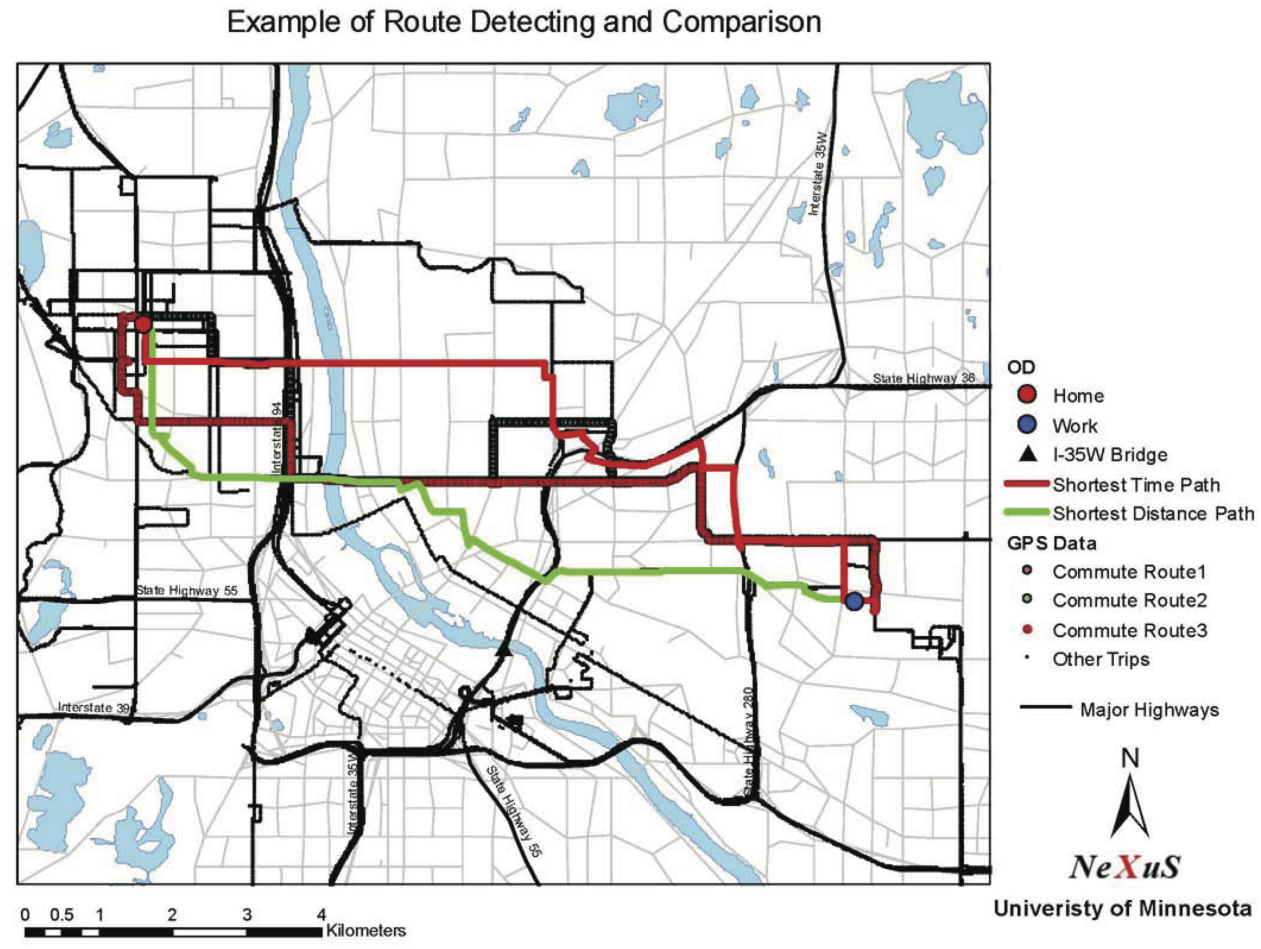
Example of commute route identification and comparison.

The GPS data provide the origin, destination, departure time and arrival time of each trip. These data, combined with the speed map we developed in the early section, allow us to evaluate the actual route choice and shortest time and distance routes (see Fig 6 for example). Both travel time of the actual route and shortest time path are evaluated based on the speed corresponding to the departure time of each trips. Any trips starting before 6:00 am or after 7:00 pm are excluded from this study because we do not have enough speed data to support such analysis.

In total 25,157 trips conducted by 143 of the study’s subjects have been identified by applying filtering criteria discussed in this section. Among them, 6,059 are commute trips. It should be pointed out that if people stopped for more than 5 minutes on their way home or to work, these trips are broken into two trips and no longer treated as commute trips. While this treatment is consistent with our definition of trips, their impacts should be carefully evaluated in future study.

For each OD pair, the shortest distance and shortest time route have been estimated by using the speed estimation during the corresponding time of the day and time periods. These speeds are averages of the direct observations obtained from the trajectory of probe vehicles, which distinguishes this study from previous studies exclusively based on assignment models.
